# The phantoms of a high-seven - or - why do our thumbs stick out?

**DOI:** 10.1186/s12983-015-0117-x

**Published:** 2015-09-15

**Authors:** Joost M. Woltering, Axel Meyer

**Affiliations:** Chair in Zoology and Evolutionary Biology, Department of Biology, University of Konstanz, Universitätsstraße 10, 78457 Konstanz, Germany

**Keywords:** Thumb, Pentadactyly, Tetrapod, Fin, Limb, Digit, Pre-axial, Post-axial

## Abstract

The earliest tetrapods had hands and feet with up to eight digits but this number was subsequently reduced during evolution. It was assumed that lineages with more than five digits no longer exist but investigations of clawed-frogs now indicate that they posses a rudimentary or atavistic sixth digit in their hindlimb. A recent reevaluation of the stem tetrapod *Ichthyostega* predicts that its seven digits evolved from two different types of ancestral fin radials, pre-axial and post-axial. In this context we now ask the question, should we consider a pre-axial origin of the thumb as reason for its unique genetic signature?

## Introduction

When the first tetrapods emerged from the water around 400 million years ago [1] their hands and feet looked quite different from the ones seen in modern day species. Instead of the five fingers and toes characteristic for ourselves and most other extant tetrapods, the hands and feet of stem tetrapods such as *Ichthyostega* and *Acanthostega* numbered up to seven or eight digits [2, 3]. For millions of years to follow, tetrapods had six digits until this changed to the canonical pentadactyl *Bauplan* at the end of the Devonian around 350 MYA [3–5] (a period whose tetrapods remain poorly known due to fragmentation of the fossil record [6]). This organization into a limb with five digits has proven extremely stable. Reductions are quite common (as in horses, pigs and birds) but supernumerary digits, beyond the “5”, are exceedingly rare and are only known from mutant or highly inbred domesticated animals [7].

## Main text

**Fig. 1:**
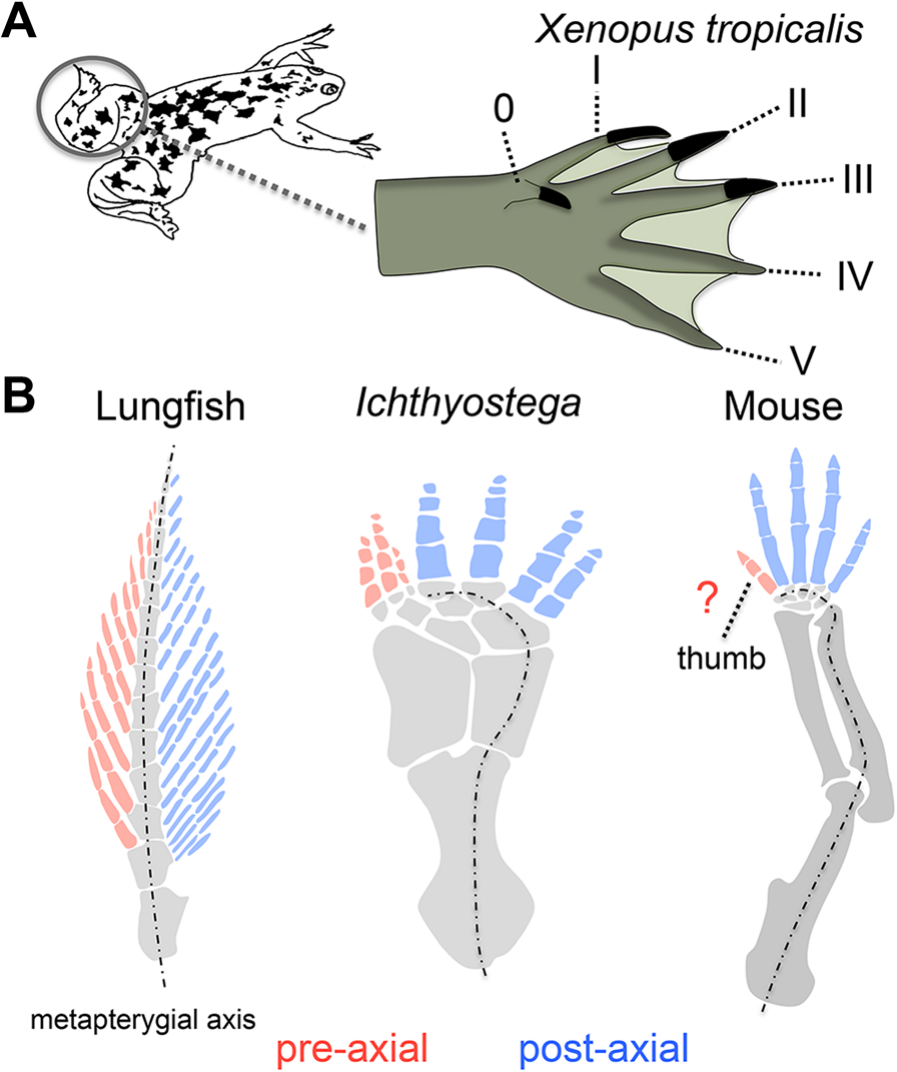
Digit 0 in *Xenopus tropicalis* and putative relationships between digits and pre-axial and post-axial sides of the fin. **a** The left hindlimb of *Xenopus tropicalis* drawn after reference [8] with indication of the digit numbers. Digit 0 appears as an antero-ventral protrusion bearing a distinct claw. (Claws on digit 0-III are drawn in black). **b** Pectoral fin of lungfish and limbs of *Ichthyostega* and mouse. The metapterygial axis is indicated with a dashed line and runs through the post-axial part of the limb in *Ichthyostega* and mouse. Mednikov [13] recently hypothesized that the three most anterior digits in *Ichthyostega* derived from the pre-axial side of ancestral fins (indicated in red). Given the unique genetic position of the thumb, its identity as either ‘post-axial’ (blue) or ‘pre-axial’ (red) digit could be investigated

This reign of pentadactylism now may need to be reconsidered. The lab of Koji Tamura reinvestigated the morphology of the hindlimbs in clawed frogs (*Xenopus tropicalis)* and they suggest that a well developed claw anterior to the thumb is in fact a true digit [8]. This would mean that frogs (at least on their feet) have six digits (of which the authors name the first one digit 0, as not to interfere with the classical patterning of I-V from thumb to pinky finger) (Fig. 1a). The occurrence of anterior, digit like structures (going by the names of pre-pollux and pre-hallux) was already well-documented [9], but these were generally assumed to be modified carpal bones. This assumption however needs to be placed in the historical context that for a long time five was considered the archetypical number of digits. It is now becoming increasingly clear that the ancestral number of digits is higher than five [2, 3, 5, 10] and this realization could ignite a new debate on whether the pre-pollux and pre-hallux are digits or part of the wrist/ankle bones, in favor of an interpretation of them being true digits.

The suggestion to consider the pre-pollux/pre-hallux as true digits is not new; Galis et al. [7] already list a number of criteria to consider them as digits equal to all other digits and many 19^th^ and early 20^th^ century descriptions of the limb also classify them as digits (see references in [7]). One possible problem with this interpretation is that their ontogeny departs from that of the other five digits. The skeletal elements in the limb display a pattern of sequential splitting and branching of cartilage condensations, during which a sequence of ‘parent’ and ‘progenitor’ elements can be distinguished. These processes have been recognized over a century ago (e.g. work by I.I. Smalhausen) and have been described in an influential model by Shubin and Alberch in 1986 [11]. Although it is now clear that the ‘branching’ and ‘splitting’ events observed during chondrification do not provide a mechanism for the formation of the different limb bones (see discussion by Cohn et al. [12]), they provide a phylogenetically conserved pattern that could identify groups of limb elements sharing ontogenetic programs and evolutionary histories. The meta-analysis of the chondrification pattern in the limb suggests that branching events originating from the posterior part of the limb (emanating from the ulna/fibula and forming the ‘digital arch’) are related to the canonical five digits [11]. The pre-pollux/pre-hallux however, appear to derive from the cartilage condensation on the anterior side of the limb, that is, the radius/tibia, which would argue against their classification as digits. These observations can however be reconciled when we consider the limb in comparison with the fins of lobe-finned fish (sarcopterygians) and in light of a recent reinterpretation of the limbs of *Ichthyostega* [13], which suggests that digits can derive from anterior condensations as well.

The fins of the Australian lungfish (*Neoceratodus forsteri*) (our closest living ‘fish’ relative) are constructed as a bi-serial fin with radials present on either side of a central axis, the metapterygium, or metapterygial axis [11, 14] (Fig. 1b). These radials are referred to as pre-axial and post-axial radials. Most interpretations of the relationships between sarcopterygian fins and tetrapod limbs accept that the metapterygial axis runs through the ulna/fibula (i.e. the posterior part of the limb) [11, 12] and that digits correspond to some type of post-axial radial [14, 15] (Fig. 1b) (which possibly evolved during the fin-to-limb transition through acquisition or elaboration of a distal phase of *Hox* gene expression [16–20]). In this interpretation the radius/tibia corresponds to a pre-axial radial. As the pre-pollux/pre-hallux (or digit 0 that is) derives from the radius/tibia [11], this digit would in fact constitute a pre-axial digit, different in its origin from the post-axial digits. A recent reinterpretation of the stem tetrapod *Ichthyostega* [13] indeed suggests that the presence of pre-axial digits is an ancestral tetrapod character. *Ichthyostega* possesses seven digits, which show a clear morphological differentiation into anterior and posterior digits [2, 3, 5]. Mednikov shows that *Ichthyostega* can be interpreted as having a bi-serial limb in which the three anterior digits correspond to pre-axial digits and the four posterior ones to post-axial digits [13] (Fig. 1b). This interpretation indicates the presence of both pre-axial and post-axial digits as ancestral for tetrapods.

Could this fresh view on frog feet and stem tetrapod toes change the way we see our own extremities? The answer is, perhaps. If we look at the thumb - digit I- in the context of pre-axial versus post-axial digits, it is striking that numerous characters set it apart from the four posterior digits (i.e. digit II-V). For instance, the signaling molecule sonic hedgehog (*SHH*) is required for the formation of all the digits except digit I [21, 22] (likewise the radius does not require *SHH* whereas the ulna does [23]). Furthermore, there is a suite of genetic markers that distinguish digit I from the posterior digits [24] such as absence of *Hoxd9* through *Hoxd12* [19, 25, 26] and *dHand2* [24], and a known *Hoxd* enhancer (island II) appears to specify a distinct territory in the posterior digits excluding the thumb [27]. Regarding the thumb’s ontogeny it has been noted that the connection to the digital arch is not obvious in all species [7, 11]. Further there is a strong correlation between congenital ‘pre-axial’ radial deficiencies and thumb agenesis [28]. Also morphometric analysis suggests that the morphology of digit I evolves largely independent from the modularized behavior of the posterior digits [29] (although in this latter case arguably selection for the opposable thumb may play a significant role and morphometric analysis should be carried out across a wider range of tetrapods).

## Conclusions

Given the above considerations, the possibility exists that our thumb stands out (or sticks if you will) from the other four digits because it may share the genetic program with the pre-axial side of the ancestral fins and therefore possibly descends from a pre-axial radial (although homology amongst digits [10, 19, 30, 31] and across the fin-to-limb transition [17, 18] remain complex issues). Such hypothesis would require a careful comparison of the genetic programs of pre-axial and post-axial radials in relation to the digits. *In vivo* studies of the extinct tetrapod ancestors are obviously not possible. Their closest living approximation is the Australian lungfish (*Neoceratodus forsteri*), the only extant fish with an elaborate bi-serial fin. It will be interesting to see how the genetic programs compare in pre-axial versus post-axial radials, using for instance comparative transcriptomics. An expansion of the posterior limb field during the fin-to-limb transition [32] has recently been proposed based on shark fins, which exhibit a broader domain of what could correspond to a ‘pre-axial’ limb field (as indicated by *Alx4*, *Hand1*, *Pax9*) and a smaller domain of what would arguably resemble a ‘post-axial’ limb field (as indicated by *Hand2* and data already available for *Hoxd12* [33]). The analysis of the expression boundaries of these and other relevant genes relative to the metapterygial axis in lungfish would reveal how the regulatory programs of our limbs compare to the pre-axial and the post-axial sides of sarcopterygian fins. Considering the recent decline of the Australian lungfish in the wild and the closure of the breeding colony at the McQuairie University [34], it is uncertain if there will ever be a chance to conduct such experiments.
